# Bacterial Tolerance and Bioleaching in the Presence of Chloride

**DOI:** 10.3390/ma18184407

**Published:** 2025-09-21

**Authors:** Narine Vardanyan, Anna Khachatryan, Zaruhi Melkonyan, Nelli Abrahamyan, Sona Barseghyan, Ruiyong Zhang, Arevik Vardanyan

**Affiliations:** 1 SPC “Armbiotechnology” NAS of Armenia, 14 Gyurjyan Str., Yerevan 0056, Armeniazaruhi.melqonyan@gmail.com (Z.M.); abrahamyan.nelly95@gmail.com (N.A.); sonabars777sb@gmail.com (S.B.);; 2 State Key Laboratory of Advanced Marine Materials, Institute of Oceanology, Chinese Academy of Sciences, Qingdao 266071, China; 3 Key Laboratory of Marine Environmental Corrosion and Biofouling, Institute of Oceanology, Chinese Academy of Sciences, Qingdao 266071, China; 4 Guangxi Key Laboratory of Marine Environmental Science, Institute of Marine Corrosion Protection, Guangxi Academy of Sciences, Nanning 530007, China

**Keywords:** iron-oxidizing bacteria, sulfobacilli, chloride tolerance, adaptation, pyrite and chalcopyrite, bioleaching

## Abstract

Chloride ions can enhance the bioleaching of copper minerals, yet most biomining microorganisms are highly sensitive to chloride and cannot survive or colonize mineral surfaces in saline environments. Chloride tolerance varies among acidophilic iron-oxidizing bacteria, but the concentrations at which they remain active are generally too low to permit the industrial use of seawater. Therefore, identifying highly chloride-tolerant leaching microorganisms and studying their bioleaching potential in chloride-containing systems is of utmost importance. This study investigated chloride tolerance and adaptability of bacteria from different genera, with a focus on *Sulfobacillus thermosulfidooxidans* subsp. *asporogenes* 41, a moderately thermophilic strain that can oxidize both Fe (II) and reduced inorganic sulfur compounds (RISCs). This dual activity makes it advantageous for bioleaching by facilitating sulfur removal, generating acidity, and preventing mineral passivation. Comparative experiments on the bioleaching of pyrite and chalcopyrite demonstrated that adaptation to 0.3 M NaCl enhanced the chloride tolerance of *S. thermosulfidooxidans* subsp. *asporogenes* 41. The adapted strain exhibited significantly improved copper extraction under saline conditions compared with the native culture. Maximum copper recovery was achieved at 0.4 M NaCl, highlighting the potential of chloride-adapted moderate thermophiles for biomining applications in saline environments. In contrast the minimal inhibitory concentration for *Acidithiobacillud ferrooxidans* Dr was 0.005 M (causing 41.2% inhibition), while *Leptospirillum ferriphilum* CC was unaffected by lower concentrations (0.01–0.02 M) and only showed severe inhibition (86.5%) at 0.1 M NaCl, defining its minimal inhibitory concentration (MIC) at 0.05 M.

## 1. Introduction

Bioleaching is the method of extracting metals from secondary waste or intractable ores by the activity of leaching microorganisms [1,2,3]. Bioleaching has been used extensively to solubilize and extract a variety of metals, including copper, zinc, uranium, cobalt, nickel, and others, because of its cost-effectiveness, straightforward procedure, relatively gentle conditions, and environmentally friendly benefits [4]. This approach is significantly less harmful to the environment than conventional smelting, as it operates at ambient temperature and pressure, drastically reduces the emission of sulfur dioxide and other greenhouse gases, and consumes less energy [5]. Its role is becoming increasingly vital in supporting circular sustainability, ensuring reliable access to critical metals required for renewable energy technologies. Significant research has been dedicated to enhancing bioleaching efficiency. Investigated methods include the addition of silver ions (Ag^+^) [6,7], activated carbon [8], chloride ions (Cl^−^) [9], and the surfactant Tween-80 [10], as well as adjustments to pH and redox potential [11,12]. Among these, the introduction of sodium chloride (NaCl) is particularly noteworthy. Its use addresses the critical challenge of freshwater scarcity by enabling the potential use of alternative saline water sources in biohydrometallurgical processes [13].

Biomining microorganisms are highly sensitive to the presence of chloride ions [14,15]. The degree of chloride tolerance differs among genera and species of acidophilic bioleaching bacteria [16,17,18]. *Acidithiobacillus ferrooxidans*, the most commonly applied bacterium in bioleaching processes, has been reported to withstand only up to 6 g/L (~100 mM) of sodium chloride (NaCl) [15]. In contrast, *Leptospirillum ferriphilum* exhibits greater tolerance, with minimum inhibitory concentrations of 225 mM (~13 g/L) and 150 mM (~9 g/L) NaCl [19]. Similarly, inhibition of growth was observed at approximately 12 g/L chloride in a batch culture dominated by *L. ferriphilum* [20].

Moderate thermophiles such as *Sulfobacillus thermosulfidooxidans* have been shown to possess higher tolerance to NaCl compared with mesophilic iron-oxidizing bacteria commonly applied in mining operations [15,17], making them advantageous for bioleaching processes involving saline waters. The dual capacity of *S. thermosulfidooxidans* to oxidize both Fe^2+^ and RISCs enhances its suitability for heap bioleaching, since RISC oxidation contributes to the removal of excess sulfur species and the generation of required acidity. In addition, a halotolerant *Sulfobacillus* sp. TPY strain, isolated from hydrothermal vents, demonstrated resistance to NaCl concentrations of up to 2% (*w*/*v*) (20 g/L) [21].

Previous studies have described several mechanisms underlying chloride toxicity in acidophilic bacteria, including osmotic imbalance, cytoplasmic acidification, and the induction of oxidative stress [22,23,24]. The inhibition of biomining microorganisms has been linked to disturbances in Fe^2+^ oxidation systems and pH regulation [20]. Due to their positive internal membrane potential [23], acidophilic bacteria experience an inward flow of chloride and protons, which decreases cytoplasmic pH, disrupts cellular homeostasis, and ultimately leads to cell death in species such as *Acidithiobacillus ferrooxidans* and *Acidithiobacillus thiooxidans* [22].

Both Gram-negative and Gram-positive acidophilic microorganisms employ various mechanisms that enable them to cope with elevated chloride concentrations. Proteomic analyses have demonstrated that exposure to chloride ions induces multiple adaptive responses in these organisms. Such responses include the accumulation of amino acids, likely serving as osmoprotectants, the expression of Ycel family proteins associated with acid- and osmotic-stress resistance, and modifications of the cell membrane. These adaptive strategies have been observed in the proteomic profiles of *Acidithiobacillus caldus* and *Acidimicrobium ferrooxidans* under NaCl stress [15].

To pinpoint the molecular basis of chloride tolerance, researchers conducted a comparative genomic analysis of various acidophilic iron-oxidizing microorganisms. This study, which included bacteria from the *Nitrospirae*, *Firmicutes*, *Actinobacteria*, and *Proteobacteria* phyla, as well as archaea from *Euryarchaeota* and *Crenarchaeota*, found that *Nitrospirae* and *Firmicutes* representatives contain genes responsible for the biosynthesis and uptake of the compatible solutes ectoine, trehalose, and potassium, key compounds in osmoregulation and salt tolerance [24,25]. It was found that exogenously added trehalose exerted a positive effect on the tolerance of archaeon *Ferroplasma acidiphilum* (*F. acidiphilum*), leading to a slight, but significant increase in MIC from 700 to 750 mM [25].

Exogenous trehalose was found to enhance the chloride tolerance of the archaeon *Ferroplasma acidiphilum* (*F. acidiphilum*), resulting in a modest but statistically significant increase in the minimum inhibitory concentration from 700 to 750 mM.

The experimental supplementation of ectoine corroborated the genomic findings, demonstrating an increased MIC for NaCl in both *L. ferriphilum* and *F. acidiphilum*. This evidence for conferred osmotolerance is instrumental for formulating chloride-based strategies to optimize mineral bioleaching. According to whole-genome sequencing and transcriptomic studies, the primary osmoadaptive strategy in *Leptospirillum* sp. involves the biosynthesis of the compatible solutes trehalose and ectoine, coupled with potassium transport [25]. Notably, although genes for potassium transporters were identified in other phyla, genes for compatible solute biosynthesis were absent.

Research demonstrates that well-known biomining type strains are unsuitable for brackish water biomining due to their inability to survive or colonize minerals in saline environments. However, halotolerant acidophiles show remarkably high chloride tolerance. Research indicates that *T. prosperus*-like strains are capable of bioleaching pyrite even in the presence of high salinity (30 gL^−1^ NaCl) [26]. Moreover, a consortium consisting of a *T. prosperus*-like strain, an *Acidiphilum*-like strain isolated from an acidic saline habitat, and *S. thermosulfidooxidans* has demonstrated significant potential for efficient biomining in chloride-rich conditions [15].

Chalcopyrite is the most prevalent copper mineral, representing approximately 70% of global copper reserves [27,28]. A key challenge in chalcopyrite bioleaching is its slow dissolution rate, which is often caused by the formation of a passivation layer on the mineral surface. Some studies have shown that higher copper recovery can be achieved by increasing the leaching temperature. For instance, Gericke et al. [29] reported that more than 98% of copper could be extracted at 70 °C using an extremely thermophilic sulfur- and iron-oxidizing microorganism. Another strategy to overcome chalcopyrite passivation is chloride-assisted leaching. Several studies have demonstrated beneficial effects of chloride on chalcopyrite bioleaching [30,31]. Bevilaqua et al. [30] observed enhanced copper dissolution from CuFeS_2_ by *A. ferrooxidans* when 100 mM NaCl (~6 g/L) was added. Similarly, the presence of 3 g/L chloride improved chalcopyrite leaching by *S. thermosulfidooxidans* [31,32].

The influence of chloride ions on the bioleaching performance of the extreme thermophilic archaeon *Sulfolobus acidocaldarius* during copper sulfide concentrate leaching has been investigated. At pH 1.5 and 1% solid content, the addition of 0.5 M and 1.0 M NaCl resulted in copper dissolution of 98% and 80% after 9 days, respectively, which increased to nearly 100% and 90% after 21 days [33]. Further studies examined chalcopyrite bioleaching by *S. acidocaldarius* at chloride concentrations up to 1.0 M and 67.5 °C. Under these conditions, copper extraction reached 100% within 14 days in the presence of the archaeon, compared with only 55% in the abiotic control containing 1.0 M NaCl [34,35].

Iron-oxidizing cultures exhibited greater sensitivity to elevated chloride concentrations compared with sulfur-oxidizing cultures [17]. The study demonstrated that a chloride-tolerant sulfur-oxidizing microbe, operating alone, was more critical for maximizing chalcopyrite dissolution than a mixed consortium of iron- and sulfur-oxidizers [36].

Microbial leaching of FeS_2_ has been widely employed as a crucial pretreatment step for gold extraction [37]. Studies have shown that the presence of chloride can inhibit pyrite bioleaching by *S. thermosulfidooxidans* [38,39]. Interestingly, while NaCl addition reduced iron oxidation activity and overall FeS_2_ bioleaching, a concentration of 0.2 M appeared to promote the attachment of *S. thermosulfidooxidans* to pyrite surfaces compared with the attachment in the absence of NaCl [38,39]. Bioleaching of FeS_2_ with a mixed culture dominated by *L. ferriphilum*, *A.caldus*, *Acidimicrobium* sp., and *Sulfobacillus* sp., was inhibited by NaCl concentration at 4 g/L [40]. The presence of 7 or 20 g/L NaCl resulted in an extended lag phase for pyrite dissolution [15]. Furthermore, the study revealed that after 14 days of bioleaching in the presence of 7 or 20 g/L NaCl, only *S. thermosulfidooxidans*, a *Thiobacillus prosperus*-like (*Acidihalobacter*) strain, and an *Acidiphilum*-like strain were detected from the inoculum [15].

The bioleaching performance of a constructed consortium, comprising *Leptospirillum ferriphilum*, *Acidithiobacillus caldus,* the archaeon *Ferroplasma thermophilum*, and the halotolerant *Sulfobacillus* sp. TPY, was evaluated on chalcopyrite under NaCl stress. The investigation revealed that *Sulfobacillus* sp. TPY exhibited a high NaCl tolerance (2% *w*/*v*), in contrast to the other consortium members, which were inhibited at concentrations greater than 0.35%. The constructed microbial consortium demonstrated effective chalcopyrite bioleaching in the presence of NaCl. Copper recovery reached a maximum of 85% at 0.5% NaCl, representing a 41.7% improvement compared with the control without NaCl. However, copper extraction declined at NaCl concentrations above 0.5%, decreasing to 70% when the NaCl level reached 2% [21]. Chloride media show better results in leaching processes to treat chalcocite [41]. Nevertheless, the chloride concentrations that enhanced bioleaching were relatively low and insufficient for the direct use of seawater in mining operations. Consequently, the search for highly chloride-tolerant leaching microorganisms and investigations into metal sulfide bioleaching under chloride-rich conditions have been major research foci over the past decades [15,42,43,44]. In this context, comparative studies on chloride tolerance and adaptability of bacteria from different genera from laboratory collections were carried out. The effect of NaCl concentration on the ability of the most tolerant moderately thermophilic bacterium, *S. thermosulfidooxidans* subsp. *asporogenes* 41, to leach pyrite and chalcopyrite was evaluated.

## 2. Materials and Methods

### 2.1. Bacteria and Growth Conditions

Iron-oxidizing mesophilic bacteria *Acidithiobacillus ferrooxidans* Dr (PV731527), moderate thermophiles *Leptospirillum ferriphilum* CC (OM272948), and *Sulfobacillus thermosulfidooxidans* subsp. *asporogenes* 41 (AF137503), all isolated from acid mine drainage (AMD) in Armenia, were cultivated in Mackintosh (MAC) [45] medium containing 4 g/L Fe^2+^ as the energy source. For *S. thermosulfidooxidans* subsp. *asporogenes* 41, the medium was additionally supplemented with 0.02% yeast extract. Cultures were incubated under shaking conditions (Orbital-Shaker-Incubator ES-20/60, Biosan, Riga, Latvia) at 120 rpm and at 30, 40, and 45 °C, respectively. The medium pH was adjusted to 1.8–1.9 with 10 N H_2_SO_4_. Adaptation procedures were carried out by multiple subculturing of bacterial strains in MAC medium with Fe^2+^ in the presence of gradually increasing concentrations of NaCl.

### 2.2. NaCl Tolerance

Experiments investigating the effect of chloride ions on iron oxidation by native and adapted strains of *A. ferrooxidans* Dr, *L. ferriphilum* CC, and *S. thermosulfidooxidans* subsp. *asporogenes* 41 were conducted in 250 mL flasks containing 100 mL of MAC medium with 4 g/L Fe^2+^. Sterile 1 M NaCl solution was added to achieve final concentrations in the range from 0.005 M to 0.3 M. Flasks were inoculated with culture liquids of native or adapted bacterial strains at a concentration of 5% grown in MAC medium with Fe^2+^. The flasks were placed on a rotary shaker operating at 120 rpm and maintained at the optimal temperature for each strain. Cell number was determined using a Toma counting chamber (Glaswarenfabrik Karl Hecht GmbH & Co KG, Bavaria, Germany) at ×1000 magnification (depth 0.1 mm). Fe^2+^ oxidation activity of the strains was determined by titration with 0.01 N Ethylenediaminetetraacetic acid (EDTA) [46]. Aliquots (typically 0.5–1.0 mL of leachate) were quantitatively diluted to 50 mL with DI water to bring iron into the 5 mg/L working range. A total of 20% C_7_H_6_O_6_S (5-sulfosalicylic acid) (20% solution; 2–3 drops) served as the indicator. The solution was heated to 60–70 °C and titrated visually with 0.01 N EDTA, observing a color change from red–violet to lemon–yellow. After adding a small amount of K_2_S_2_O_8_, the solution was titrated again until the same color transition was achieved. The calculation was performed using Equation (1):

(1) Xmg/L=V1×M×56×1000/V

where *V*1—titrated volume of EDTA, *M*—molarity of EDTA (0.005 M or 0.01 N); 56—atomic mass of Fe; *V*—sample volume.

Inhibition of Fe^2+^ oxidation was determined by comparing the amount of iron oxidized in the control (without NaCl) to that in samples containing varying NaCl concentrations. All experiments were conducted in triplicate.

### 2.3. Bioleaching of Pyrite and Chalcopyrite

A comparative study was conducted on the bioleaching behavior of pyrite (FeS_2_) and chalcopyrite (CuFeS_2_) in the absence and presence of NaCl. Pyrite (FeS_2_), consisting of 43.8% Fe and 49% S, and chalcopyrite, consisting of 33.7% Cu, 26.2% Fe, and 38% S, from the copper mine of Armenia, ground to ≤ 63 µm, were used for the bioleaching experiment. For the experiments, 250 mL Erlenmeyer flasks were used, each containing 50 mL MAC medium supplemented with 0.02% yeast extract and mineral substrates (2.5 g [5%] FeS_2_ and 5.0 g CuFeS_2_). The inoculum was prepared according to the method detailed in Section 2.1. Native culture and adapted to 0.3 M NaCl culture of *S. thermosulfidooxidans* subsp. *asporogenes* 41 in a concentration of 5% (*v*/*v*) were used (about 10^7^–10^8^ cells/mL) for bioleaching of FeS_2_. Additionally, NaCl in concentrations of 0.1, 0.2, and 0.4 M was added to the appropriate flasks to study its effect on bioleaching of FeS_2_ by *S. thermosulfidooxidans* subsp. *asporogenes* 41. The experiments were conducted at 45 °C under shaking conditions (120 rpm). Liquid samples were collected periodically to measure Fe^2+^ and Fe^3+^ concentrations produced during bacterial decomposition of FeS_2_. The Fe^2+^ and Fe^3+^ ions were quantified by titration with 0.01 N EDTA, while copper released from CuFeS_2_ was analyzed using an atomic absorption spectrometer (AAS, SP-IAA1800) (Bioevopeak, Jinan, China, wavelength λ = 324.754 nm (Cu hollow cathode lamp); lamp current = 4.0 mA; slit = 0.5 nm (instrument default), pyrolytically coated graphite tubes, and a matrix-matched temperature program (drying, pyrolysis, atomization, and cleaning) were used).

For each concentration of NaCl, a corresponding control without bacteria was set. Bioleaching experiments were run in triplicate.

## 3. Results and Discussion

### 3.1. Effect of NaCl on Iron Oxidation

**Figure 1:**
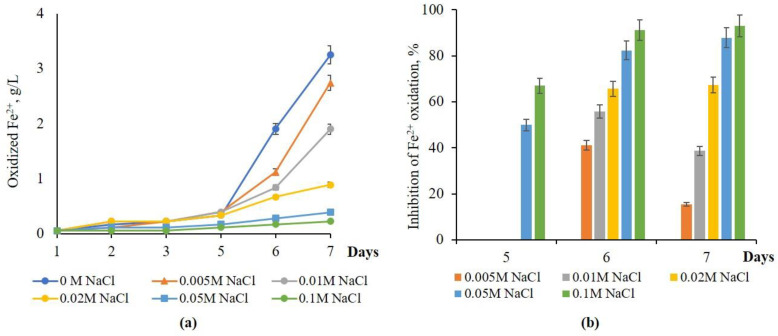
(**a**) The effect of NaCl concentration on Fe^2+^ oxidation; (**b**) Fe^2+^ inhibition by *At. ferrooxidans* Dr (30 °C, pH 1.98, 120 rpm, NaCl—0.005–0.1 M).

Growth of *A. ferrooxidans* Dr and oxidation of Fe^2+^ was observed on the 6th day of incubation, regardless of the absence and presence of NaCl (Figure 1a). The half maximal inhibitory concentration (IC_50_) of NaCl for *A. ferrooxidans* Dr was 5 mM, which caused 41.2% inhibition of iron oxidation. Moreover, the degree of Fe^2+^ oxidation inhibition increased with rising NaCl concentrations in the medium. Specifically, inhibition ranged from 41.2% to 91.2% as the NaCl concentration increased from 5 mM to 0.1 M over 6 days. These data for 7 days consisted of 15.5% and 93.1%. It is worth mentioning that the extent of inhibition of Fe^2+^ oxidation on the 7th day decreased from 41.2 to 15.5% and from 55.8 to 38.7% at 5 and 10 mM of NaCl, respectively, while inhibition of Fe^2+^ oxidation remained unaltered (constant) at concentrations of NaCl of 0.02–0.1 M (Figure 1b).

**Figure 2:**
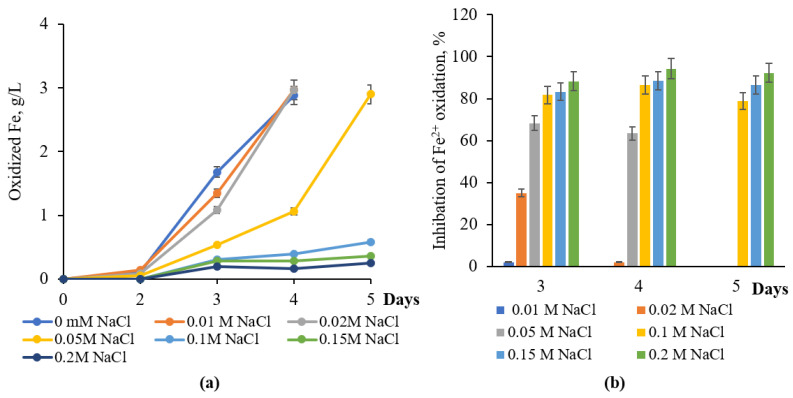
The influence of NaCl concentrations on Fe^2+^ oxidation (**a**) and inhibition (**b**) by *L. ferriphilum* CC (40 °C, 120 rpm, NaCl-concentration range: 0.01–0.2 M).

Since no significant inhibition of Fe^2+^ oxidation by *L. ferriphilum* CC was observed at 0.01 or 0.02 M NaCl during 4–5 days of bacterial growth, the NaCl MIC for *L. ferriphilum* CC can be considered 0.05 M (Figure 2a,b). This concentration caused 68.3 and 63.5% inhibition of iron oxidation by bacteria for 3 and 4 days of growth, respectively (Figure 2b). However, it should be noted that bacteria completely oxidized Fe^2+^ at 0.05 M NaCl for 5 days (Figure 2a,b). In the presence of 0.1 M or higher NaCl concentrations in the medium, a pronounced inhibition of iron oxidation was observed. Thus, extents of iron oxidation inhibition were 86.5% and 78.9% at 0.1 M and 94.2% and 92.3% at 0.2 M NaCl for 4 and 5 days of growth, respectively (Figure 2b).

**Figure 3:**
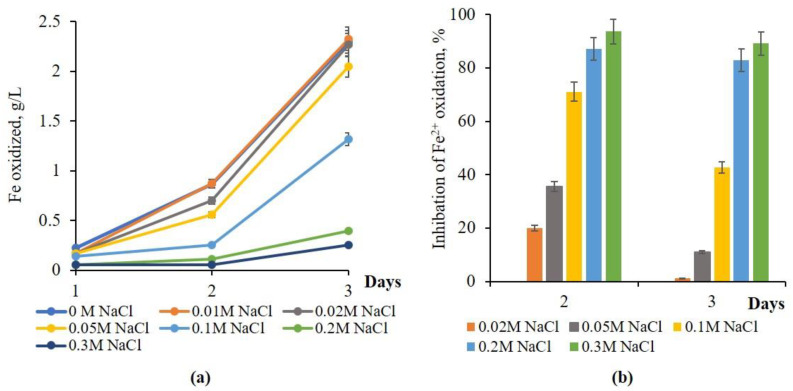
The influence of NaCl concentrations on Fe^2+^ oxidation (**a**) and inhibition (**b**) by native culture *S. thermosulfidooxidans* subsp. *asporogenes* 41 (45 °C, 120 rpm, NaCl-concentration range: 10–300 mM, this study).

The NaCl MIC for native culture *S. thermosulfidooxidans* subsp. *asporogenes* 41 was shown to be 0.05 M, which caused 35.3% inhibition of Fe^2+^ oxidation on the 2nd day of growth and only 11% inhibition on the 3rd day. At concentrations of NaCl of 0.2 and 0.3 M, from 83 to 94% of the iron oxidation activity of *S. thermosulfidooxidans* subsp. *asporogenes* 41 was detected regardless of growth duration. In the case of 0.1 M NaCl, 71% suppression of iron oxidation by *S. thermosulfidooxidans* subsp. *asporogenes* 41 was observed on the 2nd day, which decreased to 42.7% on the 3rd day (Figure 3b). No detectable inhibition of iron oxidation by *S. thermosulfidooxidans* subsp. *asporogenes* 41 was observed throughout the experiment at 0.01 and 0.02 M NaCl (Figure 3b).

The majority of investigations into chloride tolerance among acidophiles have been carried out with *At. ferrooxidans* and *At. thiooxidans* [14,26,47,48,49]. Our results on *A. ferrooxidans* tolerance are fully consistent with data obtained by other authors [14,18,42,47,50], confirming that NaCl in a concentration of 6 g/L (about 0.1 M) completely inhibits the growth of *A. ferrooxidans*. The findings suggest that the native strain *L. ferriphilum* CC has a higher NaCl MIC compared to *A. ferrooxidans*. Consistent with this, several researchers have reported that *L. ferriphilum* tolerates NaCl more effectively, with MIC values of 225 mM (~13 g/L) and 150 mM (~9 g/L) [19,20,25]. MIC for NaCl obtained for *S. thermosulfidooxidans* 41 is equal to that observed for *Sulfobacillus acidophilus*—1 mg/L (0.017 M) [32]. In contrast, *Sulfobacillus acidophilus* DSM 10332 strain Cutipay, recovered from a naturally harsh mining environment in Northern Chile, demonstrated a higher minimum inhibitory concentration of 5 g/L (0.085 M). This strain demonstrated enhanced copper recovery and high potential for chalcopyrite bioleaching [32]. Another strain, *Sulfobacillus* sp. TPY was able to tolerate 2% (*w*/*v*) NaCl, whereas the other three microorganisms were inhibited when NaCl concentrations surpassed 0.35% [21]. Therefore, our research confirmed the consideration that moderate thermophilic *S. thermosulfidooxidans* is more tolerant to NaCl, which makes it beneficial for bioleaching in saline water [15]. The enhanced NaCl tolerance observed in the moderate thermophile *S. thermosulfidooxidans*, compared to the mesophiles, is consistent with findings from comparative genomic studies. These studies suggest that certain thermophilic iron oxidizers within the *Firmicutes* phylum (which includes *Sulfobacillus*) may harbor genetic determinants for the biosynthesis and uptake of compatible solutes, such as ectoine and trehalose, as well as potassium transporters [19,25]. While this study did not quantify solute accumulation or gene expression, the successful phenotypic adaptation of *S. thermosulfidooxidans* subsp. *asporogenes* 41 to 0.3 M NaCl aligns with the proposed role of such mechanisms in halotolerance. The absence of these genes in many mesophilic acidophiles could explain their inherently lower chloride tolerance. Therefore, the adaptive response we observed may involve the upregulation of these very pathways, a compelling hypothesis for future genomic or proteomic verification.

### 3.2. Iron Oxidation Bacteria Adapted to NaCl

**Figure 4:**
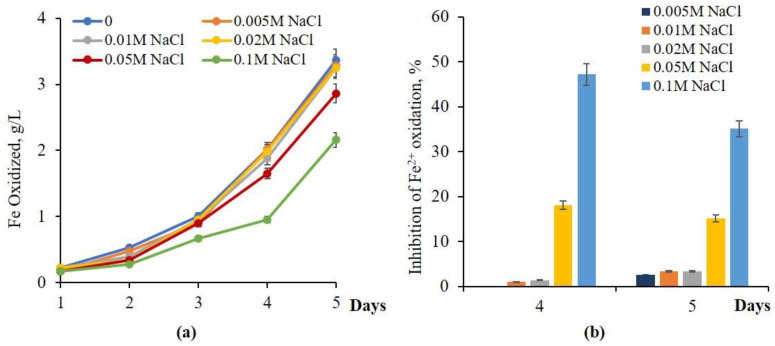
Influence of NaCl on Fe^2+^ oxidation (**a**) and inhibition (**b**) by adapted (0.02 M NaCl) culture *At.ferrooxidans* Dr (30 °C, pH 1.86, 120 rpm, NaCl-concentration range: 0.005–0.1 M).

According to Figure 4, adapted to a 0.02 M NaCl culture, *A. ferrooxidans* Dr showed improved oxidation activity in comparison with the native culture. It should be noted that the adapted culture showed almost no inhibition of iron oxidation at NaCl concentrations of 0.02 M or lower (Figure 4a). Only 18% and 47.2% inhibition were observed at 0.05 and 0.1 M NaCl for 4 days of growth of adapted culture *At.ferrooxidans* Dr. It is worth mentioning that, along with growth inhibition of Fe^2+^, oxidation decreased to 15 and 35% at 0.05 and 0.1 M NaCl, respectively (Figure 4b).

**Figure 5:**
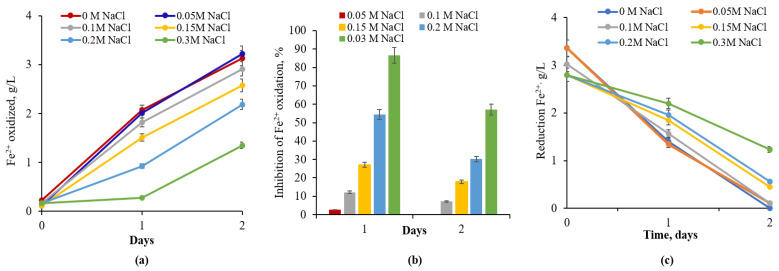
The influence of NaCl concentration on Fe^2+^ oxidation (**a**), inhibition of Fe^2+^ oxidation (**b**), and remaining Fe^2+^ concentration (**c**) by the adapted culture of *S. thermosulfidooxidans* subsp. *asporogenes* 41 (45 °C, 120 rpm, NaCl-concentration range: 0.05–0.3 M).

NaCl MIC for adapted culture *S. thermosulfidooxidans* subsp. *asporogenes* 41 increased about 2 times from 0.05 to 0.1 M (Figure 5a,c). This concentration led to only 12.3 and 7.2% inhibition of iron oxidation by bacteria for 1 and 2 days of growth, respectively. Iron oxidation inhibition by the adapted culture at 0.2 M NaCl on the second day was 30.2%, which was significantly less than that observed by the native culture of *S. thermosulfidooxidans* subsp. *asporogenes* 41 at the same concentration for the same period of growth (87.1%). Moreover, iron oxidation by the adapted culture at 0.3 M NaCl was inhibited only by 57.1% for 2 days (Figure 5b).

### 3.3. Bioleaching of Pyrite with and Without NaCl

**Figure 6:**
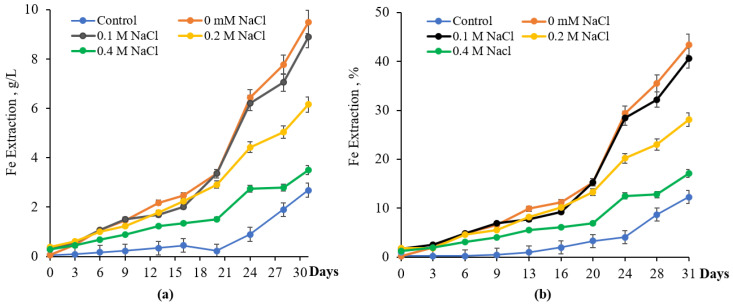
Extraction of iron g/L (**a**) and % (**b**) during bioleaching of FeS_2_ by native culture of *S. thermosulfidooxidans* subsp. *asporogenes* 41 (control-without bacteria, PD-5%, pH 1.88, t-45 °C, 120 rpm).

Comparative experiments were performed to assess pyrite bioleaching by native and adapted cultures of *S. thermosulfidooxidans* subsp. *asporogenes* 41. Data presented in Figure 6 showed that 0.1 M NaCl had a negligible effect on bioleaching of pyrite (Figure 6).

Thus, the amounts of iron extracted from pyrite by the native culture of *S. thermosulfidooxidans* were nearly identical whether NaCl was absent or at 0.1 M, and exceeded the uninoculated control by approximately 3.3–3.5 times. In contrast, the Fe released dropped from 9.5 g/L to 6.2 g/L and 3.75 g/L under 0.2 M and 0.4 M NaCl, respectively. In general, inhibition of iron bioleaching for 31 days at 0.1, 0.2, and 0.4 M NaCl comprised 2.8, 15.3, and 26.3%, respectively, compared to the option without NaCl (Figure 6b).

**Figure 7:**
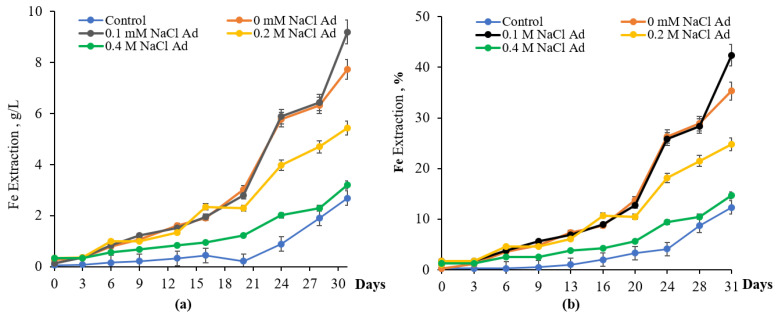
Extraction of iron g/L (**a**) and % (**b**) during bioleaching of FeS_2_ by adapted culture *S. thermosulfidooxidans* subsp. *asporogenes* 41 (control-without bacteria, PD-5%, pH 1.88, t-45 °C, 120 rpm).

Results in Figure 7 showed that when previously adapted to 0.3 M NaCl culture *S. thermosulfidooxidans* subsp. *asporogenes* 41 was used, the amount of iron released from FeS_2_ was 7.7, 9.2, 5.4, and 3.2 g/L without NaCl and in the presence of 0.1, 0.2, and 0.4 M NaCl in the medium. Therefore, the extent of inhibition of pyrite bioleaching in the presence of 0.2 and 0.4 M NaCl was significantly less compared with the native culture *S. thermosulfidooxidans* subsp. *asporogenes* 41 and 10.5 and 20.6% in comparison to the option without NaCl. Moreover, some stimulation (about 7.1%) of pyrite bioleaching by adapted culture was observed at 0.1 M NaCl for 31 days (Figure 7).

These results are consistent with literature data that pyrite dissolution declined as the NaCl concentration increased [15,39,40]. However, their data indicate that 0.1 M NaCl suppressed both iron oxidation and the growth of *S. thermosulfidooxidans* during pyrite bioleaching.

### 3.4. Effect of NaCl on Chalcopyrite Bioleaching

**Figure 8:**
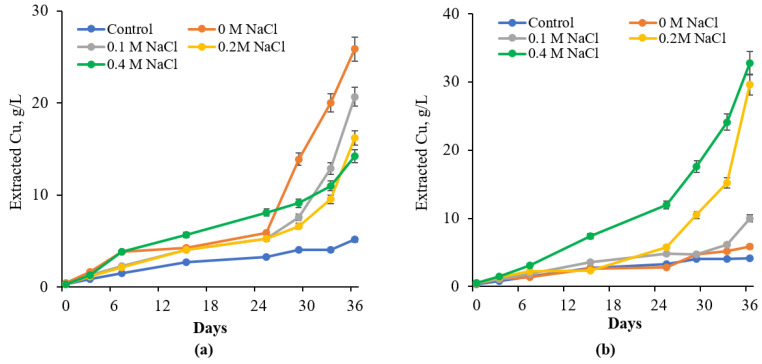
Influence of NaCl on recovery of copper from chalcopyrite by native (**a**) and adapted cultures *S. thermosulfidooxidans subsp. asporogenes* 41 (**b**).

Comparative studies were conducted to evaluate the effects of varying NaCl concentrations on the bioleaching of chalcopyrite (CuFeS_2_) by native and adapted cultures of *S. thermosulfidooxidans* subsp. *asporogenes* 41 (Figure 8).

In the case of the native culture, the maximum amount of Cu (25.88 g/L) was observed in the medium without NaCl. Furthermore, it was observed that copper extraction decreased with increasing NaCl concentration. Consequently, copper recovery after 36 days was 20.68 g/L and 16.2 g/L in the presence of 0.1 M and 0.2 M NaCl, respectively. Increase of Cu extraction at 0.4 M NaCl could be explained by some promotion of chemical oxidation of CuFeS_2_ with NaCl (Figure 8). Recovery of Cu from CuFeS_2_ by adapted culture *S. thermosulfidooxidans* subsp. *asporogenes* 41 was 32.8, 29.58, and 10.0 g/L at 0.4, 0.2, and 0.1 M NaCl, respectively, against 5.83 g/L, observed in the absence of NaCl for 36 days (Figure 8). Thus, NaCl in concentrations 0.2 and 0.4 M led to an enhancement of Cu extraction by the adapted culture *S. thermosulfidooxidans* 41 about 5 to 8 times (Table 1). The data obtained were comparable to those reported by other authors. According to the literature, bioleaching of copper sulfide concentrate by the extreme thermophile *Sulfolobus acidocaldarius* reached approximately 100% dissolution after 14 days in 0.5 M NaCl [33].

According to Table 1, native culture *S. thermosulfidooxidans* subsp. *asporogenes* 41 exhibits maximum recovery of copper (64%) under NaCl-free conditions. With increasing NaCl levels, a decrease in the extent of copper recovery is observed. Conversely, the adapted culture showed a gradual increase in copper extraction from chalcopyrite as NaCl levels increased in the medium. Thus, a significantly higher amount of copper leached from CuFeS_2_ is detected at 0.2 M NaCl by adapted culture *S. thermosulfidooxidans* subsp. *asporogenes* 41 (73.2%) compared to native culture (40%) in the same condition. Recovery of copper by adapted bacterial culture reaches the maximum extent at a concentration of 0.4 M NaCl in the medium (nearly complete recovery). Comparative bioleaching capacity was described for *S. thermosulfidooxidans* strain Cutipay (DSM 27601) [31,32]. In another example, the marine acidophilic halotolerant bacterium *Sulfobacillus* sp. TPY tolerated 2% (20 g/L) NaCl, whereas *L. ferriphilum* and *Ferroplasma acidophilum* were inhibited at concentrations exceeding 0.35% [21,51]. Several other reports confirm that chloride ions can positively influence copper bioleaching by iron- and sulfur-oxidizing bacteria, including *S. thermosulfidooxidans* [33,36,41]. About 97% of copper was extracted from chalcopyrite in 1.0 M NaCl using *Sulfolobus acidocaldarius*, according to previous studies [34,35]. The comparative analysis of NaCl tolerance among *A. ferrooxidans*, *L. ferriphilum*, and *S. thermosulfidooxidans* highlights critical trends in bioleaching under saline conditions, with implications for industrial applications (Table 2).

As shown in Table 2, comparative analysis of NaCl tolerance reveals *S. thermosulfidooxidans* as the most promising candidate for saline bioleaching, with adapted strains achieving 100% copper recovery from chalcopyrite, highlighting the latter’s unique potential for saline bioleaching applications.

The observed divergent impacts of chloride on pyrite versus chalcopyrite bioleaching by the adapted *S. thermosulfidooxidans* strain imply the involvement of distinct mechanistic pathways. For pyrite, the inhibition is primarily likely due to the direct toxic effects of Cl^−^ on the iron-oxidation machinery of the bacteria, as evidenced by our Fe^2+^ oxidation assays (Figure 3, Figure 4 and Figure 5), resulting in a lower oxidative capacity and slower dissolution rates.

In contrast, the enhanced leaching of chalcopyrite, particularly at 0.4 M NaCl, indicates that the adapted strain can not only tolerate but thrive in these conditions to facilitate dissolution. The analysis revealed that the adapted culture maintained a high oxidizing potential throughout the experiment at 0.4 M NaCl, whereas the ratio plummeted in the native culture. This sustained regeneration of Fe^3+^ is critical for efficient chalcopyrite leaching. We propose that this, combined with the well-documented ability of chloride ions to form soluble complexes with copper (e.g., CuCl_2_^−^) [30,41], prevents the formation of surface-passivating films on the mineral. While direct mineralogical evidence (e.g., XRD/SEM–EDS) for the absence of jarosite or elemental sulfur passivation layers is beyond the scope of this study and remains a target for future work, the high copper recovery and solution chemistry data strongly support a mechanism where chloride tolerance and chloride-mediated chemistry synergistically enhance chalcopyrite bioleaching.

The adaptation of *S. thermosulfidooxidans* subsp. *asporogenes* 41 to 0.3 M NaCl and its high activity at 0.4–0.5 M NaCl presents a promising opportunity for biomining in arid, coastal regions where seawater or brackish water must be used. However, translating these flask-based results to an industrial heap or column system involves several considerations. Firstly, biofilm formation, which is crucial for efficient heap bioleaching, may be influenced by chloride ions. Interestingly, some studies suggest chloride can enhance bacterial attachment to pyrite [38,39], a phenomenon we also observed preliminarily. Secondly, the use of saline solutions introduces challenges such as potential corrosion of irrigation infrastructure and changes in solution chemistry that could affect permeability and channeling within the heap. Finally, the excellent gas transfer in shake flasks is not representative of a full-scale heap, where oxygen and CO_2_ availability often limit microbial activity. The performance of this chloride-adapted consortium under the suboptimal gas transfer conditions of a column reactor is a critical next step for validation and is the focus of our ongoing research.

## 4. Conclusions

MIC of NaCl for *A. ferrooxidans* Dr was 5 mM, which caused a 41.2% inhibition of iron oxidation. No significant inhibition of Fe^2+^ oxidation by *L. ferriphilum* CC was found at 0.01 and 0.02 M NaCl, and consequently, NaCl MIC for *L. ferriphilum* CC was considered to be 0.05 M. When NaCl reached 0.1 M or above, a pronounced 86.5% inhibition of iron oxidation was observed. The NaCl MIC for native culture *S. thermosulfidooxidans* subsp. *asporogenes* 41 was shown to be 0.05 M, which caused only 35.3% inhibition of Fe^2+^ oxidation. Thus, among the tested cultures, *S. thermosulfidooxidans* subsp. *asporogenes* 41 demonstrated significantly more tolerance to NaCl compared to *A. ferrooxidans* and *L. ferriphilum*. Thus, according to their tolerance to NaCl, the tested bacteria can be ranked in the following order: *S. thermosulfidooxidans* subsp. *asporogenes* 41 > *L. ferriphilum* CC > *A. ferrooxidans* Dr. Adaptation of bacteria in the presence of gradually increased concentration of NaCl allowed for improvement of their tolerance to some extent, depending on bacterial species. MIC of NaCl for adapted culture *A. ferrooxidans*, Dr increased about 10 times and reached 0.05 M versus 0.005 M in the case of native culture. NaCl MIC for adapted culture *S. thermosulfidooxidans* subsp. *asporogenes* 41 increased twice from 0.05 to 0.1 M and led to only 7.2% inhibition of iron oxidation by bacteria for 2 days of growth. Comparative studies on bioleaching of pyrite by native and adapted culture of *S. thermosulfidooxidans* subsp. *asporogenes* 41 showed that 0.1 M NaCl had a negligible effect on bioleaching of pyrite. Inhibition of pyrite bioleaching by native culture *S. thermosulfidooxidans* subsp. *asporogenes* 41 at 0.2 and 0.4 M NaCl comprised 15.3 and 26.3%, respectively. Adaptation of bacteria to 0.3 M NaCl allowed for a decrease in inhibition degree at the same concentration of NaCl to 10.5 and 20.6%. Moreover, at 0.1 M NaCl, about 7.1% stimulation of pyrite bioleaching by the adapted culture was observed. Adaptation of *S. thermosulfidooxidans* subsp. *asporogenes* 41 to 0.3 M NaCl led to an enhancement of Cu extraction about 5 to 8 times compared to native culture in concentrations of 0.2 M and 0.4 M NaCl, respectively. Recovery of copper by adapted culture *S. thermosulfidooxidans* subsp. *asporogenes* 41 reached the maximum extent at a concentration of 0.4 M NaCl in the medium (nearly complete recovery), sub-seawater salinity, offering a potential pathway for sustainable mining in water-scarce regions. Thus, the study provides a comprehensive assessment of NaCl tolerance in defined iron- and sulfur-oxidizing acidophiles, directly linking physiological responses to practical bioleaching performance, providing new insights for biomining in chloride-rich environments.

## Figures and Tables

**Table 1:** Recovery of copper from CuFeS_2_ by native and adapted cultures of *S. thermosulfidooxidans* subsp. *asporogenes* 41 in the presence of NaCl.

Bacterial Culture	NaCl, Concentration, M				
		0	0.1	0.2	0.4
Extracted Cu by native culture *S. thermosulfidooxidans* subsp. *asporogenes* 41	g/L	25.9	20.7	16.2	14.3
	%	64	51.2	40.0	35.0
Extracted Cu by adapted culture *S. thermosulfidooxidans* subsp. *asporogenes* 41	g/L	5.83	10.0	29.58	32.8
	%	17.3	24.7	73.2	97.3

**Table 2:** NaCl tolerance and bioleaching performance of acidophilic bacteria.

Parameter	*A. ferrooxidans*	*L. ferriphilum*	*S. thermosulfidooxidans*	Reference
Maximum NaCl tolerance (native strain)	0.1 M (6 g/L)	0.15–0.225 M (9–13 g/L)	0.05–0.1 M (2.9–5.8 g/L)	[20], this study (Figure 3)
NaCl MIC (inhibition threshold)	0.005 M (41% Fe^2+^ oxid. inhibition)	0.05 M (63–68% inhibition)	0.05 M (35% inhibition)	[19], this study (Figure 1, Figure 2 and Figure 3)
Adapted strain tolerance	0.05 M (10× improvement)	-	0.1 M (2× improvement)	This study (Figure 4 and Figure 5)
Fe^2+^ oxidation inhibition at 0.1 M NaCl	93% (native), 36% (adapted)	86.5% (native)	42.7% (native), 30% (adapted)	[14], this study (Figure 1, Figure 2, Figure 3, Figure 4 and Figure 5)
Pyrite bioleaching with NaCl	Inhibited at >0.05 M	Inhibited at >0.1 M	26% inhibition at 0.4 M (native), 20.6% (adapted)	[40], this study (Figure 6 and Figure 7)
Chalcopyrite bioleaching with NaCl	Improved at 0.1 M [30]	-	100% Cu recovery at 0.4 M (adapted)	[31], this study (Table 1)
Proposed tolerance mechanism	Osmotic imbalance, cytoplasmic acidification	K^+^ uptake, ectoine syntheses (inferred)	Compatible solutes (e.g., trehalose), membrane adaptation	[15,25]
Industrial relevance	Limited to low-salinity systems	Potential for brackish water	Best candidate for seawater bioleaching (0.4 M NaCl tolerance)	[21], this study

## Data Availability

The original contributions presented in this study are included in the article. Further inquiries can be directed to the corresponding author.

## Abbreviations

The following abbreviations are used in this manuscript:

MIC	Minimal inhibitory concentration
IC50	Half maximal inhibitory concentration
AMD	Acid mine drainage
RISCs	Reduced inorganic sulfur compounds
MAC	Mackintosh medium
EDTA	Ethylenediaminetetraacetic acid
AAS	Atomic absorption spectrometer
